# Transcriptional Regulation of Starch Biosynthesis in Sorghum Grain by a MIKC-Type MADS-Box Transcription Factor: An In Vitro Analysis

**DOI:** 10.3390/plants15071011

**Published:** 2026-03-26

**Authors:** Junkai Zhang, Zheyu Yan, Anqi Sun, Xiangling Gong, Hanmin Ma, Mingxi Huang, Yuxing Lin, Zhizhai Liu, Lanjie Zheng, Qianlin Xiao

**Affiliations:** 1 College of Agronomy and Biotechnology, Southwest University, Chongqing 400715, China; zhang_junkai0369@163.com (J.Z.); 13786041673@163.com (Z.Y.); 18090310071@163.com (A.S.); 15098024284@163.com (X.G.); 15025123719@163.com (H.M.); 18723546204@163.com (M.H.); lyx12524@126.com (Y.L.); liu003@swu.edu.cn (Z.L.); 2 College of Agronomy, Henan Agricultural University, Zhengzhou 450046, China

**Keywords:** sorghum, starch biosynthesis, MIKC-type MADS-box, transcriptional regulation, SbMIKC17

## Abstract

The MADS-box transcription factor (TF) family constitutes a critical class of transcriptional regulators in plants, playing pivotal roles in diverse developmental processes. MIKC-type proteins represent Type II MADS-box TFs that widely function in regulating floral organ development and reproductive growth in plants. In this study, a total of 38 MIKC-type MADS TFs were identified from the sorghum genome, distributed across nine chromosomes. Based on sequence alignments and phylogenetic analysis, these 38 SbMIKC genes (*SbMIKC*s) were further classified into 10 distinct subfamilies. The expression profiling of these *SbMIKC*s across multiple tissues revealed five major patterns, among which *SbMIKC17* exhibited relatively abundant transcript levels during grain development in sorghum. Further assays confirmed that the protein encoded by *SbMIKC17* localizes to the nucleus without self-transactivation activity in yeast. Integrated results from DNA affinity purification sequencing (DAP-seq), dual-luciferase assays, and yeast one-hybrid experiments demonstrate that SbMIKC17 binds to the promoter of *SbAGPS1* and activates its activity, as well as enhance the promoter activities of *SbBt1*, *SbGBSSI*, *SbSSIIa*, and *SbISA1* simultaneously. Collectively, these findings suggest that the MIKC-type MADS member of SbMIKC17 serves as a potential transcriptional regulator in starch biosynthesis in sorghum.

## 1. Introduction

Sorghum (*Sorghum bicolor* L.) is a versatile cereal crop widely cultivated in arid and semi-arid regions across the globe [1]. With an estimated annual planting area of approximately 40 million hectares and a production output of around 60 million metric tons, it ranks among the world’s top five cereal crops [1,2,3]. Like other cereals, starch is the major component in sorghum grain, constituting approximately 65% to 81% of its dry weight and existing primarily in the form of granules within the endosperm. Its composition significantly influences the physicochemical properties, production, and quality of the grain, thereby determining its diverse applications [3,4,5]. Although recent advances in synthetic biology have constructed cell-free chemoenzymatic starch biosynthesis from carbon dioxide [6], the vast majority of starch used by humans is still derived from photosynthetic processes in plants, which relies on the participation of a series of functional enzymes [7,8,9,10].

Starch biosynthesis in sorghum and other cereal crops is mediated by a coordinated array of functional enzymes [8,10]. For instance, ADP-glucose pyrophosphorylase (AGPase) catalyzes the synthesis of ADP-glucose (ADPG) and serves as the rate-limiting enzyme in starch production [11,12]. The ADPG synthesized in the cytoplasm relies on the Brittle1 (Bt1) protein for transport into the amyloplast, where it is utilized for the elongation of polyglucan chains [7,13]. Granule-bound starch synthase I (GBSSI) is responsible for the synthesis of amylose [14,15], while various soluble starch synthases (SSSs) catalyze chain elongation across different degrees of polymerization (DP) [10]. Starch branching enzymes (SBEs) facilitate the cleavage of α-1,4-glycosidic bonds and reattach the chain *via* an α-1,6-glucan linkage to form branch points [10,16]. Debranching enzyme (DBE) is not only involved in the removal of improper branch linkages but also implicated in the initiation of starch granule formation [17,18]. Starch phosphatases (SPs) have also been reported to participate in starch biosynthesis through multiple pathways [19,20]. Beyond the functional enzymes, transcriptional regulation is recognized as a pivotal mechanism governing starch biosynthesis [21,22]. For instance, several transcriptional factors (TFs), such as OsRSR1 [23], OsbZIP58 [24], OsbHLH144 [25], OsNAC20/26 [26], OsRISBZ1 [27], ZmNAC126/128/130 [28,29], ZmMYB14 [30], ZmbZIP91 [31], ZmEREB167 [32], TaNAC019 [33], and SbNAC22/68 [34,35], have been reported to participate in the regulation of starch biosynthesis.

The MADS-box TFs are widely distributed across fungi, animals, and plants [36], named for the four fabulous founder homeotic proteins, i.e., MCM1 from *Saccharomyces cerevisiae*, AGAMOUS from *Arabidopsis thaliana*, DEFICIENS from *Antirrhinum majus*, and SRF from *Homo sapiens* [37]. MADS-box genes encode a highly conserved MADS domain that facilitates DNA binding with the specific recognizing motif of CArG-box located within the *cis*-regulatory regions of target genes [36,38]. Based on the characteristics of conserved domains, plant-specific MADS-box TFs can be further classified into two types of Type I and Type II [39,40]. Type I proteins contain only the MADS (M) domain, whereas Type II proteins exhibit a characteristic MIKC structure, comprising the M, intervening (I), keratin-like (K), and C-terminal domains [39,41,42]. Hence, Type II proteins are also designated as MIKC-type MADS-box proteins [41,43], and can be further categorized into MIKC^C^ and MIKC* based on the variations within the I domain [44]. In addition, MIKC^C^-type MADS-box genes have been subdivided into a greater number of subfamilies in flowering plants, and sorghum comprises 15 of those subfamilies [45].

Plant MADS-box genes are central regulators that orchestrate multiple aspects of plant reproductive development, including flowering time, inflorescence architecture, floral organ identity, and seed development [46,47,48,49]. However, the functions of Type I MADS-box genes are primarily implicated in plant reproduction, such as female gametophyte, embryo, and seed development [50,51,52]. In contrast, MIKC-type MADS-box genes have been extensively characterized across diverse species, including *Arabidopsis*, rice, and wheat, and are established regulators of key developmental processes, notably floral morphogenesis through the ABCDE model, as well as root, embryo, and seed development and embryogenesis [48,53,54,55]. For instance, three MIKC*-Type MADS-box genes are involved in the process of pollen maturation in both *Arabidopsis* and rice [56]. SOC1, AGL15, AGL18, and AGL24 are important regulatory factors for the floral organ development [48,57,58]. LcSVP2, PlSOC1, and DAM are reported to associate with bud dormancy in evergreen perennial litchi, herbaceous peony, and European plum [59,60,61]. Multiple MIKC*-Type MADS boxes are involved in pollen maturation and tube growth in *Arabidopsis* and rice [56,62]. Additionally, MADS-box TFs are also involved in root development [63], signal transduction [64], and diverse stress responses [35]. These findings collectively demonstrate that plant MADS-box TFs possess diverse and complex regulatory functions [55,65]. However, the profiling of MADS-box genes in sorghum lags far behind that of other crops, with several documented family members and poor understanding of the potential functions.

MADS TFs have also been demonstrated in both maize and rice to function as master regulators of starch biosynthesis, critically controlling the expression of key genes and metabolic networks involved in this process. For instance, ZmES22 from maize was reported to negatively regulate starch accumulation in rice grains [66], while ZmMADS1a acts as a positive regulator for the transcription of starch biosynthesis-related genes (SBRGs) in maize [67]. The MIKC-type MADS-box protein SOC1 is considered as a prime candidate for genetic engineering aimed at boosting maize yield [68]. In rice, OsMADS14 cooperates with NF-YB1 to directly activate the expression of *OsAGPL2* and *Waxy* during the process of starch biosynthesis in rice grains [69]. Additionally, MADS29 can interact with NAC25 to modulate cytoplasmic membrane integrity, degeneration, and starch biosynthesis in rice endosperm [70]. Furthermore, the suppressing of *OsMADS7* stabilizes amylose content under high-temperature stress in rice endosperm [71]. Beyond rice and maize, the role of MADS-box TFs in governing the transcriptional regulation of genes involved in starch biosynthesis remains unelucidated in sorghum.

In the present study, we performed a comprehensive analysis using the sorghum genome (Sorghum_bicolor_NCBIv3, GCA_000003195) to elucidate the evolutionary history of MADS-box genes in sorghum and establish a foundation for functional studies of this TF family. A total of 38 MIKC-type MADS-box genes were identified and systematically characterized with respect to their phylogenetic relationships, gene structures, protein motifs, promoter *cis*-elements, gene duplication events, and chromosomal locations. Furthermore, we analyzed the expression patterns of all MIKC genes, cloned *SbMIKC17*, which is highly expressed in the endosperm, and provided preliminary evidence that it can regulate the transcription of *SbAGPS1*, a key gene involved in starch biosynthesis in sorghum grain. These findings provide a theoretical and technical basis for further functional dissection of MADS-box genes in sorghum.

## 2. Results

### 2.1. Identification of the MIKC-Type MADS Factors in Sorghum

Referring to the homologous queries of *Arabidopsis* and maize, a total of 38 MIKC-type MADS genes (*SbMIKC*s) were ultimately identified in sorghum across nine chromosomes (Chrs) except Chr05 by removing the redundant genes and alternative splicing transcripts (Table S1). The length of proteins encoded by these *SbMIKC*s ranges from 171 aa (SbMIKC12) to 476 aa (SbMIKC16), with molecular weights varying between 19.302 kDa (SbMIKC12) and 52.794 kDa (SbMIKC16) (Table S1). The isoelectric points of all SbMKKCs span from 4.98 (SbMIKC37) to 9.51 (SbMIKC3) (Table S1). Subcellular localization prediction revealed that all 38 SbMIKCs in sorghum were localized to the nucleus (Table S1).

### 2.2. Sequence Characterization of SbMIKCs

**Figure 1:**
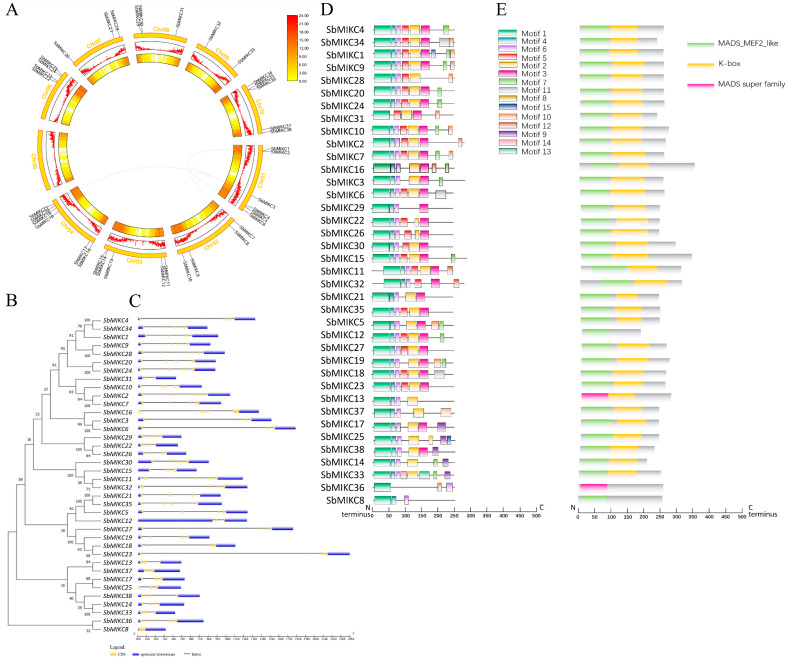
Sequence Characterization of Sorghum MIKC-Type MADS-Box Transcription Factors. (**A**) Chromosomal localization and collinearity analysis of sorghum MIKC-type MADS genes. Gray lines indicate collinear gene pairs associated with the sorghum MIKC-type MADS gene family. (**B**) Phylogenetic analysis of *SbMIKC*s. (**C**) Gene structure of *SbMIKC*s, blue and yellow boxes correspondingly represent UTRs and exons, while black lines indicate introns. (**D**–**E**) Motif composition (**D**) and domain architecture (**E**) among the proteins of SbMIKCs.

Phylogenetic trees were constructed through the methods of neighbor-joining (NJ) and maximum likelihood (ML) based on sequence alignments of 38 SbMIKCs, along with the full-length sequences of 42 MIKCs from *Arabidopsis* (AT), 44 from maize (GRMZM), and 37 from rice (LOC Os) (Figure S1). Similar results were found with both methods, and the analysis revealed that the MIKC-type MADS proteins from these four species clustered into 11 distinct subgroups. Notably, subgroup VI consisted exclusively of *Arabidopsis* members without those from sorghum, rice, and maize (Figure S1). Accordingly, MIKC-type MADS-box genes in sorghum can be classified into ten subgroups. Chromosomal localization analysis indicated that the 38 *SbMIKC*s were predominantly located near telomeric regions, which are characterized by high gene density (Figure 1A). Furthermore, collinearity analysis identified three collinear gene pairs of *SbMIKC2* (Chr01)-*SbMIKC7* (Chr02), *SbMIKC3* (Chr01)-*SbMIKC6* (Chr01), and *SbMIKC3* (Chr01)-*SbMIKC20* (Chr04) (Figure 1A).

Gene structure analysis revealed that *SbMIKC*s exhibit relatively conserved characteristics (Figure 1B). The results demonstrated that, with the exception of *SbMIKC8*, which contains only a single exon, the other 37 *SbMIKC*s typically feature multiple intron–exon structures, generally comprising 4 to 11 exons. Exon analysis indicated that *SbMIKC*s possess a relatively large exon at the 3′ end along the sequence, while the intermediate exons are comparatively smaller (Figure 1B,C). Intron analysis further showed that several *SbMIKC*s contain a relatively large first or third intron (Figure 1C).

The analysis through MEME Suite (v5.5.7) identified 15 conserved motifs among the proteins of 38 SbMIKCs. Phylogenetic clustering revealed that proteins within each subfamily displayed similar motif distribution patterns. Among these 15 identified motifs, motif 1 was shared by all 38 SbMIKCs, followed by motif 6 and motif 4, which were correspondingly shared by 37 and 36 SbMIKCs (Figure 1D). Furthermore, motifs 2/3/5/7/8 were also observed in more than 10 SbMIKCs, while motifs 9 to 15 were only sporadically distributed among a few SbMIKCs (Figure 1D).

Domain prediction using TBtools-II identified three conserved domains among the 38 SbMIKCs proteins, including MADS_MFF2-like, K-Box, and MADS super family (Figure 1E). Notably, the MADS_MFF2-like domain was present in 36 SbMIKCs except SbMIKC13 and SbMIKC36 (Figure 1E). In contrast, the MADS super family domain was primarily found in SbMIKC13 and SbMIKC36. Interestingly, SbMIKC36 and SbMIKC8 each contained only a single conserved domain: a MADS super family domain and a MADS_MFF2-like domain, respectively, and the remaining 36 SbMIKCs additionally harbored a conserved K-Box domain (Figure 1E).

### 2.3. Cis-Element Architecture and Expression of SbMIKCs

To investigate the potential functional and regulatory roles of the *SbMIKC*s, we conducted a *cis*-element analysis focused on the 2000 bp within the promoter regions upstream TSS for all 38 *SbMIKC*s. A total of 56 distinct *cis*-elements were identified, which were classified into four functional categories, including phytohormone-responsive elements, growth and development-related elements, light-responsive elements, and stress-responsive elements (Figure S2 and Table S2).

**Figure 2:**
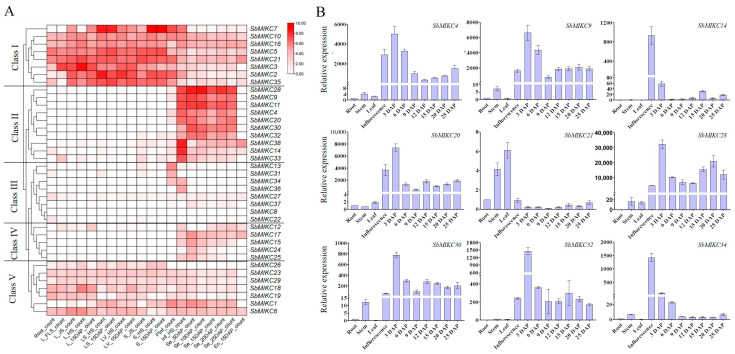
Transcriptional profiles of sorghum MIKC-type MADS-box genes. (**A**) The expression pattern and clustering analysis of *SbMIKC*s among different tissues based on the RNA-seq data. (**B**) The expression analysis of nine *SbMIKC*s among different tissues through qRT-PCR.

Based on RNA-seq data from multiple sorghum tissues [72], we analyzed the expression patterns of 38 *SbMIKC*s (Figure 2A). The results showed that *SbMIKC*s could be classified into five distinct categories of Class I to V (Figure 2A). *SbMIKC*s in Class I were expressed across all tissues examined, with higher expression levels observed in vegetative organs, such as roots, stems, and leaves, compared to floral organs and grains (Figure 2A). Members in Class II were predominantly highly expressed in floral organs and seeds, but transcripts were barely detectable in the endosperm (Figure 2A). Class III genes showed low transcript abundance across all tissues, although a few genes exhibited relatively high expression in floral organs (Figure 2A). Class IV genes exhibited moderate transcript levels in flowers, seeds, and endosperm, while their expression signals were almost undetectable in samples other than those related to inflorescence and seed (Figure 2A). Notably, *SbMIKC17* in Class IV possessed a relatively higher expression level than the other members of this group (Figure 2A). Class V genes were consistently expressed at comparable levels across all tissues (Figure 2A).

According to RNA-seq data, nine *SbMIKC*s were identified with relatively high transcript abundance and further validated by qRT-PCR across developing sorghum grains three to 25 days after pollination (3–25 DAPs), as well as floral, stem, root, and leaf tissues. The results revealed that *SbMIKC4*, *9*, *20*, *28*, *30*, and *32* exhibited higher transcript levels in inflorescences and developing grains, whereas *SbMIKC21* was more abundant in roots, stems, and leaves. In contrast, *SbMIKC14* and *SbMIKC34* showed elevated expression during early stages of inflorescence and grain development (Figure 2B).

### 2.4. Functional Properties of SbMIKC17

**Figure 3:**
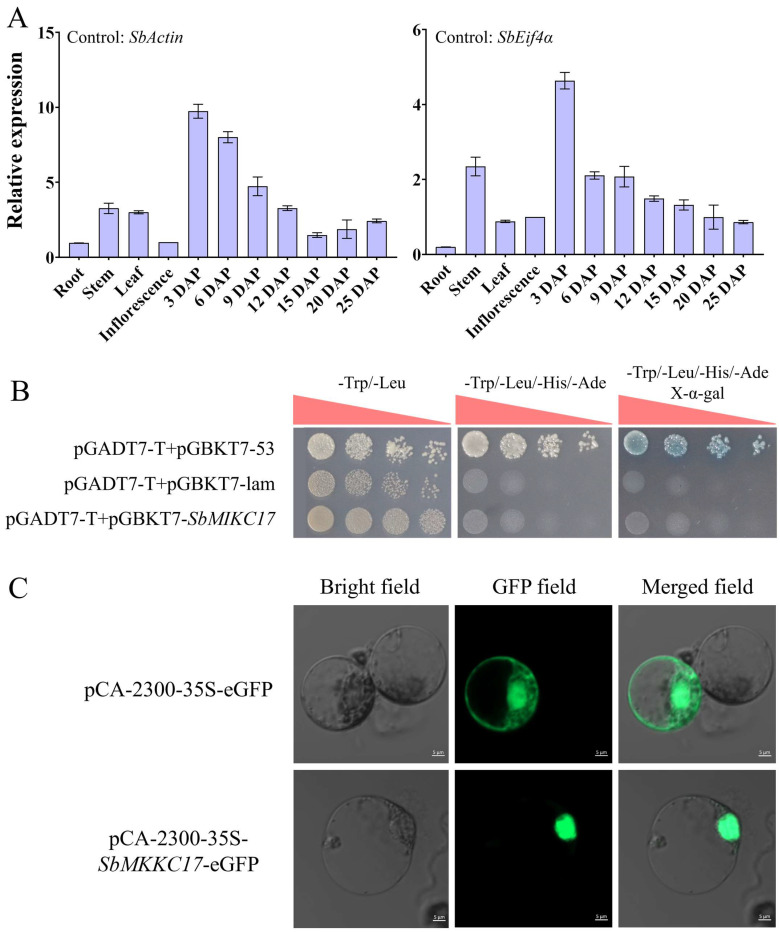
The functional properties analysis of *SbMIKC17*. (**A**) The expression pattern of *SbMIKC17* among different tissues in sorghum. (**B**,**C**) The self-activation analysis (**B**) and sub-cellular location (**C**) of SbMIKC17, white bars refer to 5 µm.

Considering the extreme inflorescence- and seed-specific expression pattern, and relative higher expression levels within these tissues, *SbMIKC17* was selected and independently characterized. The results of qRT-PCR revealed that *SbMIKC17* was highly expressed within the seed samples at 3 DAPs, while detectable transcript levels were also observed in root, stem, leaf, and floral tissues (Figure 3A). During grain development, the relative expression of *SbMIKC17* gradually decreased. Nonetheless, its expression remained generally higher in developing grains compared to that in roots and other vegetative tissues throughout various developmental stages (Figure 3A).

The transactivation activity of SbMIKC17 was assessed using the GAL4-based Yeast Two-Hybrid (Y2H) system. The recombinant plasmid pGBKT7-SbMIKC17, along with the negative control pGBKT7 and the positive control pGBKT7-ZmMYB14 [30], was transformed into the yeast strain AH109. Successful transformations were confirmed by growth on nutrient-deficient media and validated by PCR screening. Upon addition of the X-α-Gal substrate, distinct degradation patterns were observed among the sample groups: the positive control clones displayed a clear blue coloration, whereas neither the negative control nor the experimental sample showed any colorimetric reaction. These results indicate that SbMIKC17 does not possess self-activating transactivation activity in yeast (Figure 3B). Furthermore, the subcellular localization of SbMIKC17 was examined in maize leaf protoplasts. The eGFP-tagged SbMIKC17 fusion protein consistently localized to the nucleus, suggesting that SbMIKC17 functions as a nuclear protein (Figure 3C).

### 2.5. Identification of the Target Gene Regulated by SbMIKC17

**Figure 4:**
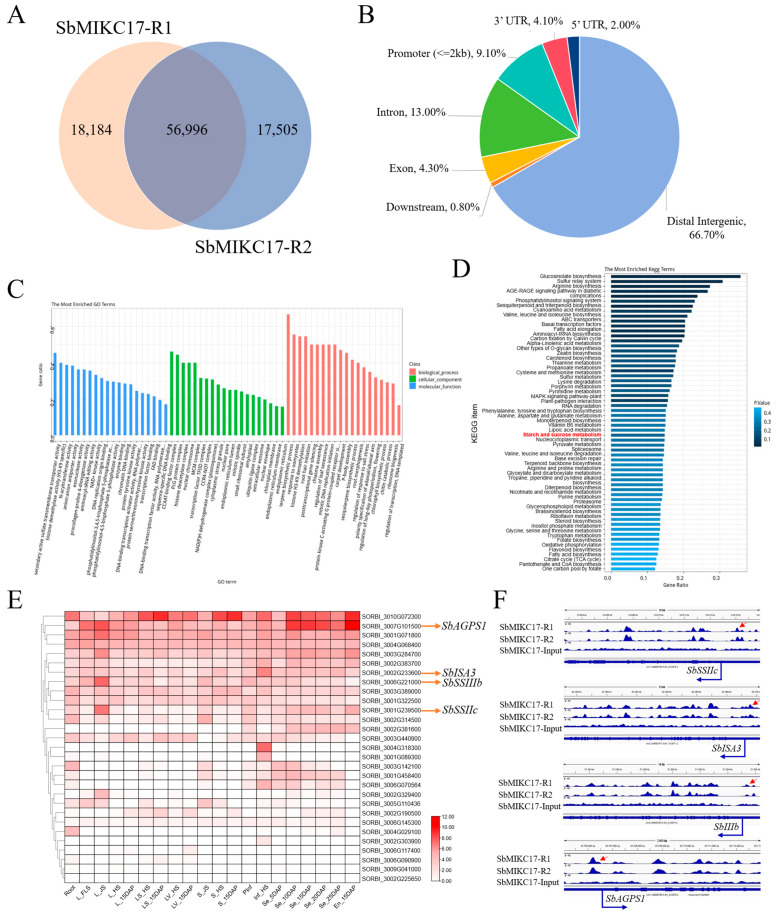
DAP-seq analysis of SbMIKC17. (**A**) Venn analysis of binding sites between two biological replicates via DAP-seq. (**B**) The genomic distribution of SbMIKC17 binding peaks identified by DAP-seq. (**C**,**D**) GO annotation (**C**) and KEGG analysis (**D**) of genes associated with the genomic loci identified through the DAP-seq analysis of SbMIKC17. (**E**) Expression analysis of key starch and sucrose metabolism genes downstream of SbMIKC17 identified by DAP-seq. (**F**) IGV screenshot showed four SbMIKC17 DAP-seq enriched peaks located in the genes of *SbAGPS1* (SORBI_3007G101500), *SbISA3* (SORBI_3002G233600), *SbSSIIIb* (SORBI_3006G221000), and *SbSSIIc* (SORBI_3001G239500).

To confirm the direct binding sites and downstream target genes of SbMIKC17, a preliminary *in vitro* analysis was conducted through DNA affinity purification sequencing (DAP-seq). A total of 56,996 shared binding sites were identified across two biological replicates (Figure 4A). Among these shared sites, 66.70% of the peaks were located in intergenic regions, while 33.30% were situated within genic regions and their flanking 2 kb regions (Figure 4B). Notably, 9.10% of the peaks were specifically distributed within the 2 kb upstream region of the TSS that correspondingly associated with 7766 genes (Figure 4B and Table S3).

GO enrichment analysis of the promoter-associated peaks revealed significant enrichments across all three major categories of biological process, cellular component, and molecular function. The most highly enriched terms within each category were terpene biosynthetic process (biological process), CCAAT-binding factor complex (cellular component), and secondary active sulfate transmembrane transporter activity (molecular function) (Figure 4C). KEGG pathway analysis indicated significant enrichment in steroid biosynthesis, sesquiterpenoid and triterpenoid biosynthesis, nucleocytoplasmic transport, and carbon metabolism (Figure 4D). Additionally, starch and sucrose metabolism was identified as another prominently enriched pathway (Figure 4D).

By integrating transcriptomic data, expression pattern analysis was performed on key genes involved in starch and sucrose metabolism that contained binding peaks in their promoter regions. Several genes exhibited relatively high transcript levels during grain development (Figure 4E). Furthermore, based on the accession numbers of key sorghum SBRGs identified in a previous study [72], *SbAGPS1* (SORBI_3007G101500), *SbISA3* (SORBI_3002G233600), *SbSSIIIb* (SORBI_3006G221000), and *SbSSIIc* (SORBI_3001G239500) were confirmed to encode critical enzymes in sorghum starch biosynthesis. Notably, binding sites for SbMIKC17 were detected in the promoter regions of all these genes (Figure 4F). Expression analysis revealed that *SbISA3*, *SbSSIIIb*, and *SbSSIIc* possessed relatively low expression levels in sorghum grains, whereas *SbAGPS1* exhibited significantly higher transcript abundance during grain development (Figure 4E). Consequently, *SbAGPS1* was identified as a candidate downstream target of SbMIKC17 in the regulation of starch biosynthesis in sorghum grains.

### 2.6. SbMIKC17 Directly Regulates the Transcription of SbAGPS1

**Figure 5:**
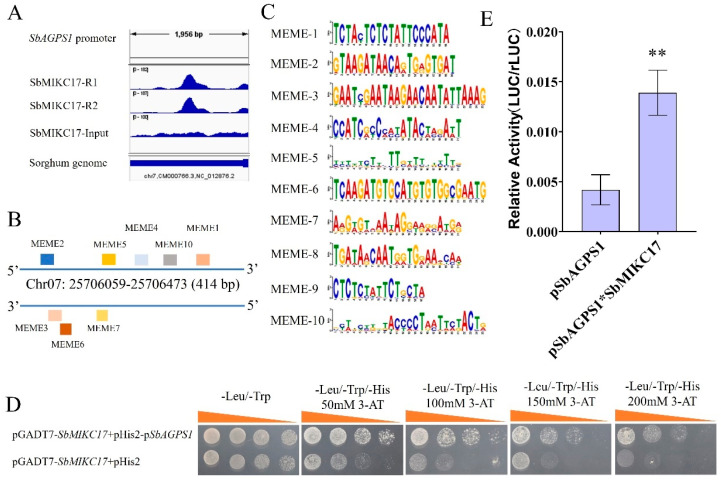
Transcriptional Regulation of *SbAGPS1* by SbMIKC17. (**A**) The IGV screenshot reveals the precise peak location in the *SbAGPS1* gene. (**B**) The genomic coordinates of the peak identified within the *SbAGPS1* promoter region, as determined by DAP-seq analysis. The differentially colored blocks represented the locations of predicted motifs across the peak interval. (**C**) Sequence information of the motifs in the peak region of *SbAGPS1* predicted by DAP-seq. (**D**) Yeast one-hybrid assay demonstrating the binding characteristics between SbMIKC17 and the promoter region of *SbAGPS1*. (**E**) The effect of SbMIKC17 on the promoter activity of *SbAGPS1*. Data are given as means and S.D.; significant differences are determined by Student’s *t*-test (*n* = 6); ** refers to the significance level of *p* < 0.01.

Through DAP-seq analysis, a 414 bp peak region was identified within the promoter region of *SbAGPS1* (Figure 5A), located between positions 25,706,059 and 25,706,673 on chromosome 7 (Figure 5B). Motif analysis revealed that this region contains 10 predicted motifs (Figure 5C), which might serve as binding sites for SbMIKC17. Meanwhile, yeast one-hybrid assays demonstrated that SbMIKC17 could directly bind to the promoter fragment of *SbAGPS1* (Figure 5D). Furthermore, dual-luciferase assays confirmed that SbMIKC17 significantly enhanced the promoter activity of *SbAGPS1* (Figure 5E).

### 2.7. SbMIKC17 Regulates the Promoter Activities of Other Key SBRGs

**Figure 6:**
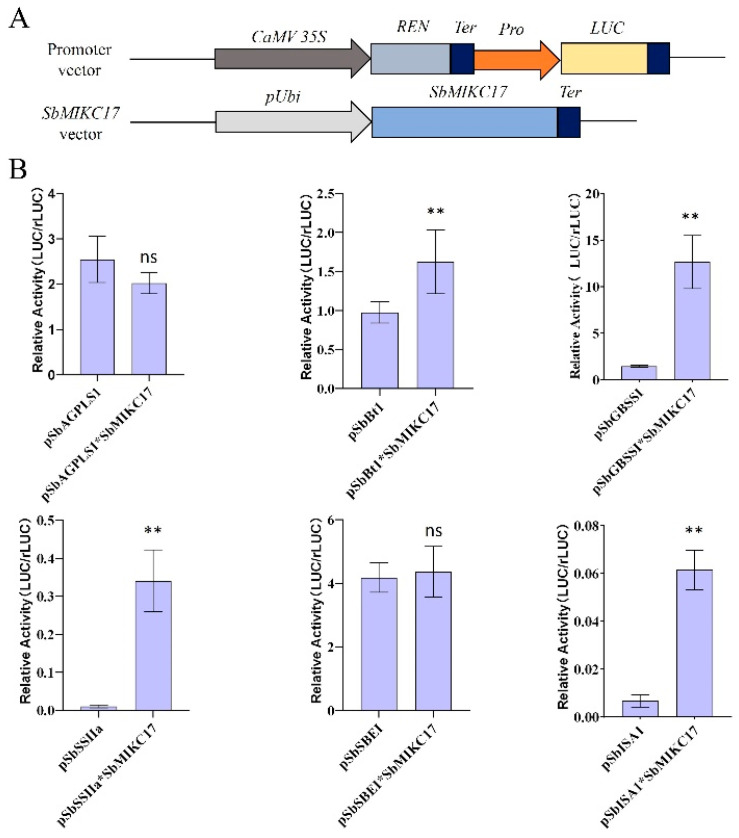
The transcription activity of other SBRGs by SbMIKC17. (**A**) Schematic of experimental vector construction. (**B**) Effect of *SbMIKC17* on promoter activity of SBRGs in sorghum, ns and ** refer to non-significance and significance at *p* < 0.01.

To further investigate the role of SbMIKC17 in the transcriptional regulation of starch biosynthesis in sorghum grains, we cloned the promoters of six SBRGs, including *SbAGPLS1* (1377 bp), *SbSSIIa* (1917 bp), *SbSBEI* (1869 bp) 81, *SbBt1* (1956 bp), *SbGBSSI* (1902 bp) [72], and *SbISA1* (1320 bp) (Figure 6A). Then, we co-transformed the *pUbi-SbMIKC17* and *pGreenII0800-Pro-Luc* constructs into maize leaf protoplasts. The activities of Renilla luciferase (rLUC) and firefly luciferase (LUC) were measured, and the LUC/rLUC ratio was calculated to assess the effect of SbMIKC17 on promoter activities of candidate SBRGs. The results demonstrated that SbMIKC17 significantly enhanced the promoter activities of *SbBt1*, *SbGBSSI*, *SbSSIIa*, and *SbISA1* in transient assays but had no significant effect on the promoters of *SbAGPLS1* and *SbSBEI* (Figure 6B). However, the underlying regulatory mechanism requires further investigation.

## 3. Discussion

The MADS-box TF family represents a crucial class of transcriptional regulators in plants, playing pivotal roles in various developmental processes [46,55,65]. Previous studies have demonstrated that MADS-box TFs are involved in the regulation of multiple stages of plant development, from seed germination to the formation of vegetative organs, and from floral to seed development [36,46,47,50,65]. Meanwhile, genome-wide identification of MADS-box family members has been accomplished in several crops, including maize [73,74], rice [47,75], *Arabidopsis* [75], barley [76], and sorghum [74]. These comprehensive and systematic identifications have laid a solid foundation for elucidating the functional diversity of MADS-box TFs in plants.

MIKC-type members are key components of the MADS-box family and participate extensively in the regulation of plant growth and development [56,59,77,78]. In this study, 38 *SbMIKC*s were identified as the candidates in the sorghum genome (Table S1). These 38 *SbMIKC*s formed 11 distinct subclades, differing in detail from those of wheat, herbaceous peony [59], and peanut [78]. Proteins encoded by these SbMIKCs possessed family-specific conserved domains and motifs, even exhibited shared spatial distribution and organizational features of the family members from other plants [59,78]. Despite overall conservation, certain variations in gene expression and sequence features were also observed among sorghum MIKC_MADS members, suggesting potential functional divergence and their ability to participate broadly in sorghum development.

The tissue-specific expression patterns of genes are closely associated with their biological functions. In sorghum, expression analysis of *SbMIKC*s also revealed considerable diversity (Figure 2A). Notably, highly expressed genes in grains and floral organs are likely involved in grain development. For instance, in rice, *MADS78* and *MADS79*, which are highly expressed in floral organs and grains, directly regulate early grain development [79]. Similarly, *PlSOC1* from herbaceous peony and *LcSVP2* from litchi, which are highly expressed in buds, also play crucial roles in bud dormancy transition [59,61]. Expression analysis of *SbMIKC*s also revealed that *SbMIKC17* and *SbMIKC38* exhibit abundant transcript levels in the endosperm, suggesting their potential involvement in metabolic regulation within this tissue (Figure 2A). Meanwhile, various *cis*-elements were identified in the promoters of *SbMIKC*s, including light-responsive, gibberellin-responsive, auxin-responsive, salicylic acid-responsive elements and defense/stress-related cis-elements (Figure S2 and Table S2), and similar *cis*-elements were also identified in the promoters of MIKC genes from other plant species [78,80]. The presence of these *cis*-elements in MIKC_MADS promoters suggests their potential roles in signal response. For example, the MIKC-type gene *MADS31* in rice was reported to be involved in salinity tolerance, and MIKC-type MADS TFs in peanut were documented to be induced by abiotic stress [78,81].

Transcriptional regulation is also an important regulatory mode for starch biosynthesis in cereals, and numerous TFs involved in this pathway were documented in rice, maize, wheat, sorghum, and other crops [21,33,34,35,70]. Among all the reported TFs, MADS-box family members have been implicated in the regulation of starch biosynthesis and yield performances of cereals and other crops, such as rice [66], maize [68,82], and blueberry [83]. In this study, preliminary DAP-seq analysis revealed that SbMIKC17 from the MIKC-type MADS family could directly bind to the promoter region of *SbAGPS1*, significantly enhancing the promoter activity (Figure 5). Furthermore, SbMIKC17 transiently activated the promoter activities of several additional candidate SBRGs (Figure 6). However, direct binding to these promoters was not confirmed, and the detailed mechanisms remain undiscovered. Collectively, our findings suggest that SbMIKC17 might serve as a potential TF involved in starch biosynthesis in sorghum grains, pending *in planta* functional validation.

## 4. Materials and Methods

### 4.1. Plant Materials and Growth Conditions

The sequenced sorghum line of BTx623 used in this study was cultivated under general irrigation and fertilization practices on the college farm land of Xiema, Beibei (29°48′ N, 106°27′ E, Chongqing, China). Five distinct tissues of root, stem, leaf, inflorescence, and grains at 3, 5, 6, 9, 12, 15, 20, 25, and 30 DAPs were sampled across the plant’s life cycle. For each tissue, three biological replicates were collected, rapidly frozen in liquid nitrogen, and stored at −80 °C for subsequent analysis.

### 4.2. Identification of MIKC-Type TFs from the Sorghum Genome

To identify putative MIKC-type TFs in the sorghum genome, a conserved domain analysis was performed using the Pfam MADS domain profile (IPR002100) to screen the *Sorghum bicolor* reference genome (v3.1.1). Candidate genes were retrieved via blastp searches against NCBI (https://www.ncbi.nlm.nih.gov/, accessed on 12 October 2025) and Gramene (https://www.gramene.org/) databases using *Arabidopsis* and maize MIKC-type sequences as queries. Putative genes were further validated with SMART (https://smart.embl.de/, accessed on 8 December 2025) and NCBI-CDD (https://www.ncbi.nlm.nih.gov/Structure/bwrpsb/bwrpsb.cgi, accessed on 8 December 2025), after which incomplete or redundant sequences were removed through alignment.

### 4.3. Bioinformatic Analysis of Sorghum MIKC-Type TFs

To elucidate the evolutionary relationships among sorghum MIKC-type TFs, a multiple sequence alignment was initially conducted using MUSCLE (v3.8.425). Neighbor-joining (NJ) phylogenetic trees were subsequently constructed in MEGA 11.0 with 1000 bootstrap replicates, employing the Poisson model and pairwise deletion of gaps [84]. In addition, the phylogenetic analysis was also performed through the maximum likelihood (ML) with the clustering method of unweighted pair group method with arithmetic mean (UPGMA). Segmental and tandem duplication events were identified using MCScanX, and syntenic relationships were visualized with TBtools-II [85]. Genomic annotation data were analyzed through TBtools-II to characterize the exon–intron structures of sorghum MIKC-type genes (*SbMIKC*s) [85]. Conserved motifs in sorghum MIKC-type TFs were identified through the online MEME suite (https://meme-suite.org/meme/, accessed on 8 December 2025), and their distributions were visualized with TBtools-II [85]. The 2000 bp genomic sequences upstream of the transcription start site (TSS) of all *SbMIKC*s were retrieved from the reference genome using TBtools-II [85]. Putative *cis*-elements in these promoter regions were predicted with the online tool PlantCARE (http://bioinformatics.psb.ugent.be/webtools/plantcare/html, accessed on 10 December 2025) and visualized using TBtools-II [85].

### 4.4. Transcriptome Data Analysis

FPKM values, retrieved from our previously published multi-tissue sorghum RNA-seq data [72], were used to analyze the expression profiles of *SbMIKC*s. Gene expression was normalized based on FPKM, and heatmaps were generated using TBtools-II [85].

### 4.5. RNA Extraction and Quantitative Real-Time PCR

Total RNA was isolated from various tissues with TRIzol reagent (Invitrogen, Carlsbad, CA, USA) and treated with DNase I (Invitrogen, Carlsbad, CA, USA) to eliminate genomic DNA contamination. First-strand cDNA was synthesized from 1.5 µg of total RNA using the PrimeScript™ RT Reagent Kit (TaKaRa, Kusatsu, Japan) according to the manufacturer’s protocol. Quantitative real-time PCR (qRT-PCR) was carried out in a 10 µL reaction mixture containing 1 µL of cDNA, using the Bio-Rad CFX96 Real-Time System. *Sorghum bicolor eukaryotic translation initiation factor 4α* (*SbEif4α*) was employed as an internal reference gene, and relative expression levels were calculated using the 2^–∆∆CT^ method normalized to *SbEif4α* expression [72]. All primers used are provided in Table S4.

### 4.6. Gene Cloning and Vector Constructions

KOD high-fidelity DNA polymerase (Toyobo, Osaka, Japan) was used to clone the *SbMIKC17*, and the PCR products were subsequently cloned into the pMD-19T vector (TaKaRa, Dalian, China). The cloned gene sequence was further confirmed by DNA sequencing. The primers used for gene cloning are listed in Table S4. The pBI221 plant expression vector was modified by replacing the original CaMV 35S promoter with the *Ubiquitin* (*Ubi*) promoter for transient overexpression assays. *SbMIKC17* was subcloned into this modified pBI221 vector under the control of the *Ubi* promoter using *Bam*HI and *Sac*I restriction sites incorporated into the PCR primers to enable directional cloning. The vector pCAMBIA2300-35S-eGFP was employed for subcellular localization studies. *SbMIKC17* was amplified via PCR using primers designed containing the restriction sites of *Bam*HI and *Xba*I without the termination codon. The amplified product was subsequently cloned into the pCAMBIA2300-35S-eGFP vector to generate a C-terminal fusion with enhanced green fluorescent protein (eGFP). A GAL4-based yeast two-hybrid system was employed to examine the self-activation activity of SbMIKC17 in yeast. *SbMIKC17* was subcloned into the pGBKT7 vector using primers containing the restriction sites of *Nde*I and *Bam*HI. All primers used for vector construction are listed in Table S4. All constructs were generated using the ClonExpress^®^ MultiS One Step Cloning Kit (Vazyme, Nanjing, China).

### 4.7. Functional Property Analysis of SbMIKC17

The subcellular localization of SbMIKC17 was analyzed in maize leaf protoplasts. The recombinant plasmid pCAMBIA2300-35S-*SbMIKC17*-eGFP was introduced into protoplasts through polyethylene glycol (PEG)-mediated transformation with Ca^2+^ co-treatment. Then, the protoplasts were incubated in darkness for 16 h to allow transgene expression prior to fluorescence imaging. Fluorescence signals were acquired using an LSM 800 confocal microscope (Zeiss, Jena, Germany) equipped with 488 nm blue light excitation.

To assess the transactivation activity of SbMIKC17, the recombinant plasmid pGBKT7-*SbMIKC17* was transformed into the yeast strain AH109. Individual transformants were cultured in 2 mL of SD/-Trp liquid medium and grown to logarithmic phase (OD_600_ = 0.6–0.8) with shaking at 150 rpm. For transcriptional activation assays, the cultures were spotted onto quadruple-dropout medium (SD/-Trp-His-Ade-Leu) supplemented with 24 µg/mL X-α-Gal, followed by incubation at 28 °C in darkness for three days. In addition, the recombinant plasmids pGBKT7-*SbMIKC17* and pGADT7-T were co-transformed into the yeast strain Y2H using the Super Yeast Competent Cell Preparation and Transformation Kit Plus (Coolaber, Beijing, China) to evaluate the transactivation activity of SbMIKC17. The plasmid combinations pGADT7-T with pGBKT7-53 and pGADT7-T with pGBKT7-lam were used as the positive and negative controls, respectively. Individual transformants were cultured in 2 mL of SD/-Leu/-Trp liquid medium and grown to logarithmic phase (OD_600_ = 0.6–0.8) with shaking at 150 rpm. For transcriptional activation assays, the cultures were spotted onto quadruple-dropout medium (SD/-Trp-His-Ade-Leu) supplemented with 24 µg/mL X-α-Gal, followed by incubation at 28 °C in darkness for three days.

### 4.8. DAP-Seq Library Preparation and Data Analysis

DAP-seq (DNA affinity purification sequencing) was performed [86,87]. Recombinant SbMIKC17 protein fused to a HaloTag was heterologously expressed using the TnT SP6 High-Yield Wheat Germ Protein Expression System (Promega, Madison, WI; L3260) and subsequently affinity-purified with Magne HaloTag Beads (Promega, G7281) in accordance with the manufacturer’s instructions. The genomic DNA (gDNA) library was prepared using the NEBNext^®^ Ultra™ II DNA Library Prep Master Mix Set for Illumina^®^ (New England Biolabs, Ipswich, MA, USA; E6040S). High-molecular-weight genomic DNA was extracted via phenol-chloroform purification and ethanol precipitation, then mechanically sheared into fragments ranging from 200 to 500 bp using the Q800R3 Sonicator (Qsonica, Newtown, CT, USA). The sonication settings were as follows: amplitude 70%, pulse rate 30 s ON/30 s OFF, total time 10 min. Sonication was performed in a 4 °C cooling water bath. End repair, dA-tailing, and adapter ligation were performed using the Hieff NGS DNA Library Prep Kit (Yeasen, Shanghai, China; 13577ES08) according to the manufacturer’s instructions.

For the binding reaction, 25 µL Magne HaloTag Beads (Promega; G7281) bound with SbMIKC1-HaloTag were incubated with 500 ng of the gDNA library in 40 µL of PBS buffer under slow rotation at 16 °C for 2 h. The beads were then washed five times with 200 µL of PBS containing 0.005% (*v*/*v*) NP-40. Bound DNA was eluted in 25 µL of elution buffer (EB, 50 mM Tris-HCl, pH 8.5) by incubation at 98 °C for 10 min. Eluted DNA was amplified with 14–17 cycles using Canace Pro Amplification Mix (Yeasen; 12624ES24) and indexed primers. The optimal concentration of the DAP-seq library for sequencing was determined based on the fragment size distribution. As a negative control, mock DAP-seq libraries were generated in parallel using the same procedure without the addition of recombinant protein.

Sequencing was performed on the Illumina HiSeq platform (150 bp paired-end). Raw data were processed with Trimmomatic v0.36 to remove low-quality reads and adapter sequences. The resulting high-quality reads were aligned to the *Sorghum bicolor* reference genome (NVBIv3; GCA_000003195.3) using BWA mem v0.7.12-r1039. Peak calling was performed with MACS2 v2.2.7.1 using parameters -g 7.3e8 -q 0.05 --nomodel --extsize 200 --keep-dup all, with mock control as background. Peaks with q < 0.05 were considered significant [88]. Target genes were defined as those harboring DAP-seq peaks within transcribed regions, 2 kb upstream of the transcription start site (TSS), 5′ UTR, exons, introns, 3′ UTR, or 2 kb downstream of the transcription termination site (TTS). The distribution of reads across gene bodies and flanking regions was analyzed using Deeptools v3.5.0. IGV_2.17.2 was employed for the visualization of peaks from DAP-seq data.

Gene Ontology (GO) enrichment analysis of differentially expressed genes (DEGs) was conducted with GOATOOLS v0.9.9. *p*-values were adjusted using the false discovery rate (FDR) method, with an FDR ≤ 0.05 considered statistically significant. KEGG pathway enrichment was performed using KOBAS 3.0, with the Benjamini–Hochberg (BH) correction applied and a significance threshold of FDR ≤ 0.05. Motif discovery was carried out with MEME v5.0.5.

### 4.9. Dual-Luciferase Reporting Assay

Dual-luciferase assays were conducted in maize leaf protoplasts using the pGreenII0800-LUC vector system, following a previously described method [72]. The coding sequence (CDS) of *SbMIKC17* was cloned into a modified plant expression vector under the control of the *Ubi* promoter. The promoter regions of sorghum starch biosynthesis-related genes (SBRGs) were inserted into the pGreenII0800-LUC vector to drive the expression of the firefly luciferase (LUC) reporter gene. Plasmid constructs were co-transformed into maize protoplasts at predetermined ratios and incubated as described by Xiao et al. [29,72]. Luminescence signals from Renilla luciferase (REN) and firefly luciferase (LUC) were measured using the Dual Luciferase Reporter Gene Assay Kit (YEASEN, Shanghai, China) and quantified with a GloMax^®^ 2020 luminometer (Thermo Fisher Scientific, Waltham, MA, USA). The Renilla luciferase activity was used as internal control, and the relative promoter activity was expressed as the ratio of LUC to REN luminescence. All experiments were independently repeated three independent biological replicates, and each replicate consisted of three technical replicates. The effect of SbMIKC17 on the promoter activity of *SBRG*s was assessed using Student’s *t*-test.

### 4.10. Yeast One-Hybrid Assay

The yeast one-hybrid assay was employed to examine the binding of SnMIKC17 to the promoter region of *SbAGPS1* (p*SbAGPS1*). A 1956 bp fragment of p*SbAGPS1* was amplified with primers containing restriction sites of *Eco*RI and *Mlu*I (Table S4) and subsequently subcloned into the yeast expression vector pHIS2 to form the p*SbAGPS1-pHis2* plasmid. Meanwhile, the coding sequence of *SbMIKC17* was amplified using primers *SbMIKC17*-Rec2F (sense) and *SbMIKC17*-Rec2R (antisense), which were designed with restriction sites of *Eco*RI and *Sac*I (Table S4), respectively. The resulting product was cloned into the pGADT7-Rec2 vector to generate the *pGADT7-Rec2-SbMIKC17* construct, which was then utilized in the yeast one-hybrid assays. The *pGADT7-Rec2-SbMIKC17* with p*SbAGPS1-pHis2* was co-transformed into the yeast strain Y187 by using the Super Yeast Competent Cell Preparation and Transformation Kit Plus (Coolaber, Beijing, China) as the experiment group, and the combinations of pGADT7-SbMIKC17 and pHis2 were used as negative controls.

### 4.11. Statistical Analysis

All experiments were performed with at least three biological replicates. Data are presented as mean ± SD. For qRT-PCR, relative expression levels were calculated using the 2^–∆∆CT^ method normalized to *SbEif4α*. For dual-luciferase assays, statistical significance was determined by two-tailed Student’s *t*-test (** *p* < 0.01). DAP-seq peaks with q < 0.05 (MACS2) were considered significant. GO and KEGG enrichment analyses were performed with FDR correction, and terms with FDR ≤ 0.05 were deemed significant. Phylogenetic analyses were conducted using both N and ML methods with 1000 bootstrap replicates in MEGA X.

## 5. Conclusions

A total of 38 MIKC-type MADS genes were identified in the sorghum genome, distributed across nine chromosomes and classified into ten distinct subfamilies. Promoter analysis revealed the presence of multiple *cis*-elements within the promoter regions of these *SbMIKCs*, and all genes exhibited five distinct expression patterns, among which *SbMIKC17* showed an inflorescence- and seed-specific expression pattern and exhibited relatively higher transcript levels during grain development and in the endosperm. *SbMIKC17* encodes a nuclear-localized protein without self-transactivation activity. Furthermore, SbMIKC17 could directly bind to the promoter of *SbAGPS1* and significantly enhance its activity. Similar functions were also observed for some other sorghum *SBRG*s, including *SbBt1*, *SbGBSSI*, *SbSSIIa*, and *SbISA1*. Collectively, these results suggest that SbMIKC17 might serve as a potential transcriptional regulator in starch biosynthesis in sorghum grains.

## Data Availability

The sequencing data can be downloaded at: https://www.ncbi.nlm.nih.gov/sra/PRJNA1416650 (accessed on 5 February 2026). Other data are contained within the present article. All other relevant data are included within the article and its Supplementary Information Files.

## Supplementary Materials

The following supporting information can be downloaded at: https://www.mdpi.com/article/10.3390/plants15071011/s1, Figure S1: Phylogenetic tree of MIKC-type MADS proteins from *Arabidopsis*, maize, rice, and sorghum. (A,B): Phylogenetic tree constructed by the method of NJ (A) and ML (B). Red triangles in both A and B refer to SbMIKC17. Figure S2: Composition of *cis*-elements within the promoters of *SbMIKC*s. Table S1: Gene information and physicochemical properties of sorghum MIKC_MADS subfamily; Table S2: Cis-elements in the 2000 bp promoter sequence of the sorghum MIKC gene via PlantCARE; Table S3: Gene information associated with the SbMIKC17 peaks identified via DAP-seq; Table S4: All the primer sequence information used in this study.
