# Supplementary figures and images for: Transcriptional Regulation of Starch Biosynthesis in Sorghum Grain by a MIKC-Type MADS-Box Transcription Factor: An In Vitro Analysis
Source: Plants (Basel). 2026 Mar 26;15(7):1011. doi: 10.3390/plants15071011 (PMC13074354; doi:10.3390/plants15071011)

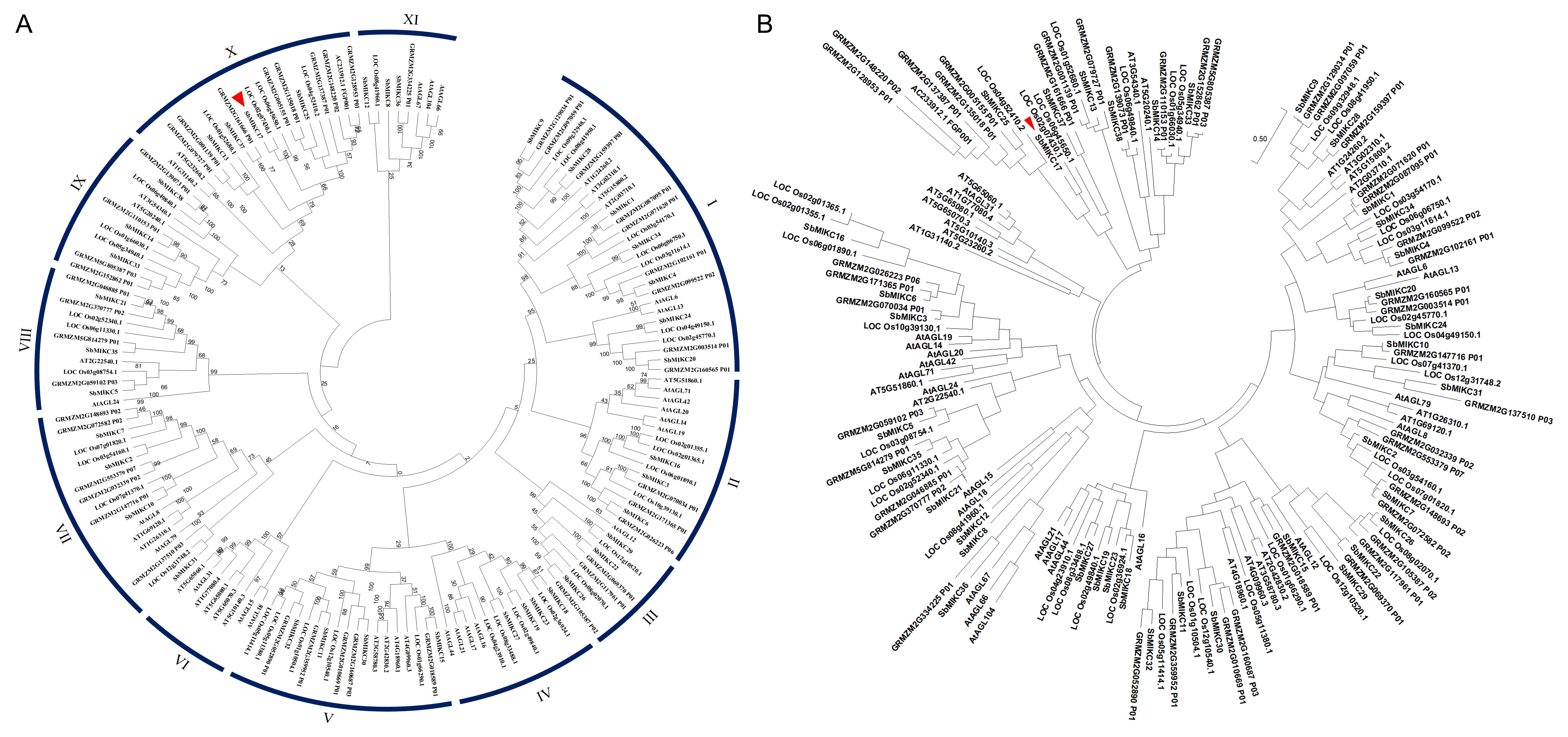

Supplement: Supplementary file 1 [file plants-15-01011-s001.zip › Fig. S1-Revised.jpg]

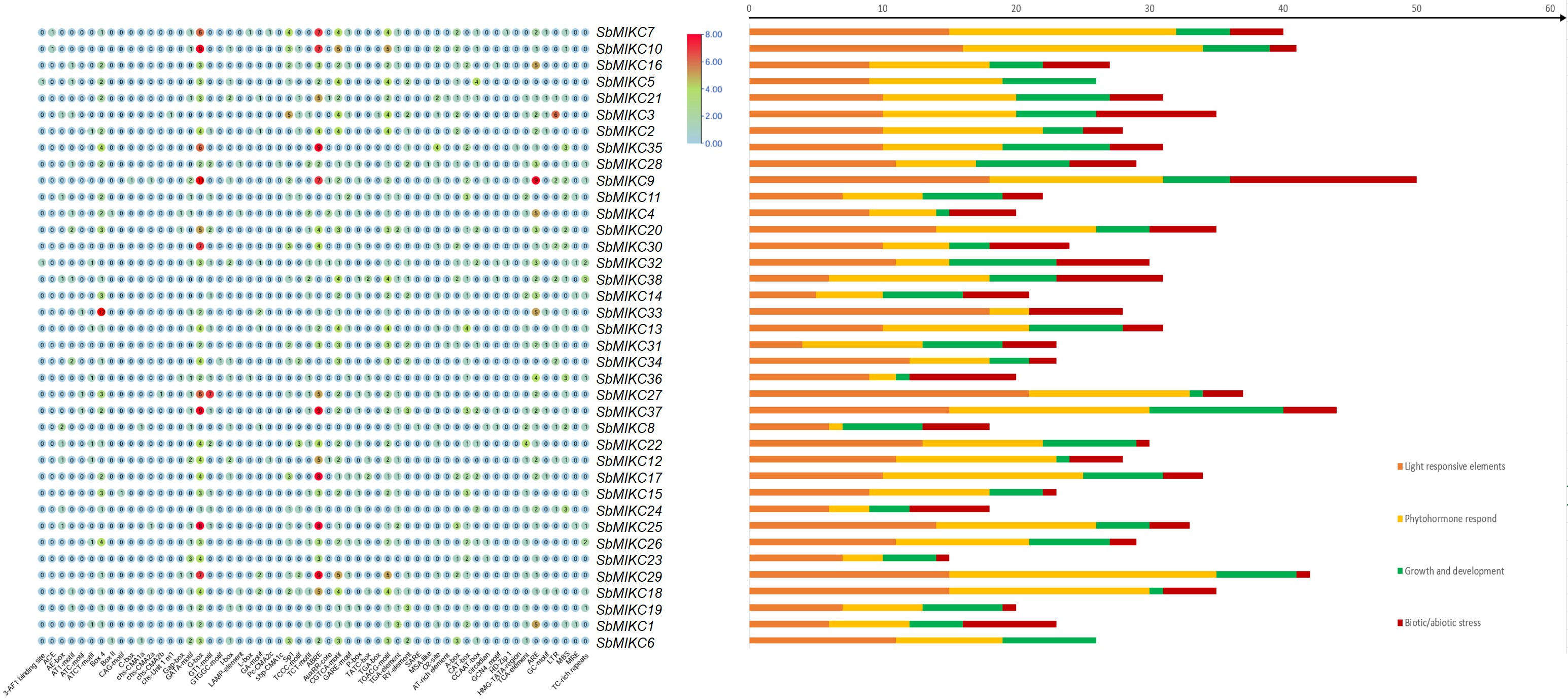

Supplement: Supplementary file 1 [file plants-15-01011-s001.zip › Fig. S2-Revised.jpg]
